# *Cryptococcus neoformans* Infection in Mice Lacking Type I Interferon Signaling Leads to Increased Fungal Clearance and IL-4-Dependent Mucin Production in the Lungs

**DOI:** 10.1371/journal.pone.0138291

**Published:** 2015-09-18

**Authors:** Ko Sato, Hideki Yamamoto, Toshiki Nomura, Ikumi Matsumoto, Tomomitsu Miyasaka, Tong Zong, Emi Kanno, Kazuko Uno, Keiko Ishii, Kazuyoshi Kawakami

**Affiliations:** 1 Department of Medical Microbiology, Mycology and Immunology, Tohoku University Graduate School of Medicine, Sendai, Miyagi, Japan; 2 Department of Science of Nursing Practice, Tohoku University Graduate School of Medicine, Sendai, Miyagi, Japan; 3 Louis Pasteur Center for Medical Research, Kyoto, Japan; Rutgers University, UNITED STATES

## Abstract

Type I interferons (IFNs) are secreted by many cell types upon stimulation via pattern recognition receptors and bind to IFN-α/β receptor (IFNAR), which is composed of IFNAR1 and IFNAR2. Although type I IFNs are well known as anti-viral cytokines, limited information is available on their role during fungal infection. In the present study, we addressed this issue by examining the effect of IFNAR1 defects on the host defense response to *Cryptococcus neoformans*. In IFNAR1KO mice, the number of live colonies was lower and the host immune response mediated not only by Th1 but also by Th2 and Th17-related cytokines was more accelerated in the infected lungs than in WT mice. In addition, mucin production by bronchoepithelial cells and expression of MUC5AC, a major core protein of mucin in the lungs, were significantly higher in IFNAR1KO mice than in WT mice. This increase in mucin and MUC5AC production was significantly inhibited by treatment with neutralizing anti-IL-4 mAb. In contrast, administration of recombinant IFN-αA/D significantly suppressed the production of IL-4, but not of IFN-γ and IL-17A, in the lungs of WT mice after cryptococcal infection. These results indicate that defects of IFNAR1 led to improved clearance of infection with *C*. *neoformans* and enhanced synthesis of IFN-γ and the IL-4-dependent production of mucin. They also suggest that type I IFNs may be involved in the negative regulation of early host defense to this infection.

## Introduction

*Cryptococcus neoformans* is a yeast-type opportunistic fungal pathogen with a capsule structure consisting of polysaccharides such as glucuronoxylomannan and galactoxylomannan, which infects lungs via an air-borne route [1]. Most healthy individuals undergo asymptomatic infection with granulomatous lesions caused by *C*. *neoformans* in the lungs, which prevents this fungal pathogen from hematogenous dissemination into extrapulmonary organs [2, 3]. However, immunocompromised hosts with severely impaired cellular immunity, such as those with hematological malignancy and acquired immunodeficiency syndrome, often suffer from disseminated infection with this fungus into the central nervous system, leading to life-threatening meningoencephalitis [4, 5].

*C*. *neoformans* is resistant to phagocytic killing by macrophages; they multiply within these cells because they have an escape mechanism that prevents them from being killed by these cells [6]. Classically activated macrophages are, however, very efficient killers of *C*. *neoformans* [7–11]. Therefore, the cell-mediated immune response, which is strictly regulated by a balance between type 1 helper T (Th1) and Th2 cells, plays a pivotal role [2, 3, 12]. Type II interferon designated as IFN-γ, which is secreted from Th1 cells, and some innate lymphocytes such as natural killer (NK) cells, NKT cells, and γδT cells [13–17], strongly induces classically activated macrophages (M1) to kill *C*. *neoformans* via a nitric oxide (NO)-dependent mechanism [10, 18, 19] and promotes the containment of this fungal pathogen within granulomatous tissues [12], leading to improvement of the infection. However, shift of the Th1-Th2 balance toward the Th2-dominant condition induces alternatively activated macrophages (M2) [10, 20, 21] and results in worsened infection with less efficient containment of *C*. *neoformans* as a result of an ameliorated granulomatous response [12].

In addition to IFN-γ, there is another type of interferon, called type I IFN, which consists of IFN-α and IFN-β [22]. Type I IFN is produced by almost all cells and triggers the activation signal via its specific receptor, IFNAR, consisting of IFNAR1 and IFNAR2 [22]. Type I IFN is well known as an anti-virus cytokine that plays a pivotal role in the elimination of viral infection [23, 24]; it also modulates the Th1-Th2 balance in viral infection [25–27]. Its role in the host defense to bacterial and fungal infection, however, has not been fully understood. Some previous investigations reported that type I IFN suppresses the host defense immune response against *Listeria monocytogenes* and *Mycobacterium tuberculosis* [27, 28] and oppositely promotes this response to infection with *Candida albicans* [29].

With this background, in the present study we examined the effect of IFNAR1 deficiency on the clearance of *C*. *neoformans* and the host immune response using a mouse model of pulmonary infection. Here, we demonstrated that a defect in IFNAR1-triggered signaling led to the accelerated clearance of *C*. *neoformans*. In addition, the IFNAR1 defect was associated with augmented Th1 responses to cryptococcal infection, as well as an increase in IL-4-dependent mucin secretion by bronchiolar epithelial cells. These findings suggest that type I IFN may negatively regulate the early host defense to pulmonary infection with *C*. *neoformans* by suppressing the Th1-mediated immune response and the mechanical barrier system at the mucosal surface in bronchi.

## Materials and Methods

### Ethics statement

This study was performed in strict accordance with the Fundamental Guidelines for Proper Conduct of Animal Experiment and Related Activities in Academic Research Institutions under the jurisdiction of the Ministry of Education, Culture, Sports, Science and Technology in Japan, 2006. All experimental procedures involving animals followed the Regulations for Animal Experiments and Related Activities at Tohoku University, Sendai, Japan and were approved by the Institutional Animal Care and Use Committee at Tohoku University (approval numbers: 2012 IDOU-124, 2013 IDOU-257, 504). All experiments were performed under anesthesia, and all efforts were made to minimize the suffering of the animals.

### Mice

IFNAR1 gene-disrupted (KO) mice were generated and established as described previously [30], and backcrossed to C57BL/6 mice for more than eight generations. Wild-type (WT) C57BL/6 mice, purchased from CLEA Japan (Tokyo, Japan), were used as controls. Male or female mice at 6 to 8 weeks of age and 16 to 24 g of weight were used in the experiments. Mice were allocated to each experimental groups randomly. All mice were kept under specific pathogen-free conditions at the Institute for Animal Experimentation, Tohoku University Graduate School of Medicine. Breeding room was managed at room temperature; 20 to 29°C, humidity; 30 to 70%, light/dark cycle; 12 hours, and water and food were given *ad libitum*. Microbial monitoring of mice were regularly carried out by the Central Institute for Experimental Animals. We took the utmost care to alleviate any pain and suffering on the part of the mice. Mice were sacrificed by cervical dislocation prior to analysis.

### *Cryptococcus neoformans*

A serotype D strain of *C*. *neoformans*, designated as B3501 (a kind gift from Dr. Kwong Chung, National Institute of Health, Bethesda, MD, USA) was used. The yeast cells were cultured on potato dextrose agar (PDA, Eiken, Tokyo, Japan) plates for 2 to 3 days before use. Mice were anaesthetized by an intraperitoneal injection of 70 mg/kg of pentobarbital (Abbott Laboratory, North Chicago, IL, USA) and restrained on a small board. Live *C*. *neoformans* (1 × 10^6^ cells) was inoculated at 50 μl into the trachea of each mouse using a 24-gauge catheter (TERUMO, Tokyo, Japan).

### Treatment with anti-IL-4 mAb

Neutralizing anti-IL-4 mAb was purified from culture supernatants of hybridoma (clone 11B11) using a protein G column kit (Kierkegaard & Perry Laboratories), and control rat IgG was purchased from ICN Pharmaceuticals, Inc. (Aurora, OH, USA). Mice were injected intraperitoneally with either Ab at 200 μg/mouse one day before and on day 0, 3, and 7 after infection. Anti-IL-4 mAb treatment reduced the level of IL-4 by more than 90% in the infected lungs compared to that in the control rat IgG-treated mice. In addition, in an *in vitro* experiment, 10 μg/ml anti-IL-4 mAb showed an approximately 50% neutralizing effect on the suppression of IL-12p40 synthesis by bone marrow-derived dendritic cells (BM-DCs) stimulated with phosphorothioated CpG1826 (100 ng/ml: synthesized by Hokkaido System Science [Sapporo, Japan]) caused by recombinant IL-4 (100 ng/ml: PeproTech, Rocky Hill, NJ, USA), and 100 μg/ml anti-IL-4 mAb completely abrogated this suppression. BM-DCs were prepared by culturing bone marrow cells from C57BL/6 mice with 20 ng/ml murine granulocyte-macrophage colony-stimulating factor (GM-CSF, Wako, Osaka, Japan) for 8 days.

### Treatment with rIFN-αA/D

Recombinant human IFN-αA/D was provided by the Nippon Roche Research Center (Kamakura, Japan). Mice received intranasal administration of 1000 IU/mouse rIFN-αA/D every day after infection with *C*. *neoformans*. rIFN-αA/D was also administered intratracheally at the time of infection.

### Enumeration of viable *C*. *neoformans*

Mice were sacrificed two and four weeks after infection, and lungs were dissected carefully and excised, then homogenized separately in 5 ml of distilled water by teasing with a stainless-steel mesh at room temperature. The homogenates, diluted appropriately with distilled water, were inoculated at 100 μl on PDA plates and cultured for 2 to 3 days before the resulting colonies were counted.

### Histological Examination

The lung specimens obtained from mice were fixed in 10% neutral buffered formalin, dehydrated, and embedded in paraffin. Sections were cut and stained with hematoxylin-eosin (H-E) or periodic acid-Schiff (PAS) stain using standard staining procedures at the Biomedical Research Core, Animal Pathology Platform of Tohoku University Graduate School of Medicine. The stained sections were observed using a Leica DM750 microscope (Leica Microsystems, Wetzlar, Germany). The photographs were taken by a Leica ICC50 HD camera and analysed by Leica LAS EZ software (Leica Microsystems). Mucin production was estimated on the PAS-stained sections as the proportion of mucin-producing bronchi relative to total bronchi. The mucin-producing bronchi were classified into three categories:-, mucin-negative bronchi; +, bronchi with mucin production in 0 to 50% of bronchoepithelial cells; ++, bronchi with mucin production in 50 to 100% of bronchoepithelial cells.

### Immunohistochemical analysis

Lung tissues were fixed in 10% neutral buffered formalin. After paraffin-embedded blocks had been cut into 5 μm sections and mounted onto slides, the specimens were deparaffinized and rehydrated. High-temperature antigen retrieval involved boiling the slides in citrate buffer (10 mM, pH 6.0) for 5 min followed by blocking with 2% rabbit serum. The samples were incubated with biotin-conjugated anti-MUC5AC mAb at a dilution of 1:100 (clone 45M1, Thermo Fisher Scientific [Lab Vision Corporation, Fremont, CA, USA]) overnight at 4°C. Endogenous peroxidase activity was blocked by treatment with 0.3% H_2_O_2_ blocking solution for 20 min. After washing, slides were incubated with Simple Stain Mouse MAX-PO (Nichirei Bioscience, Inc., Tokyo, Japan) and were then incubated with horseradish peroxidase-conjugated streptavidin (NICHIREI, Tokyo, Japan) and washed. The slides were incubated with diaminobenzidine substrate and counterstained with Carrazzi’s hematoxylin solution (Wako, Osaka, Japan).

### Extraction of RNA and quantitative real-time RT-PCR

Total RNA was extracted from the infected lungs using Isogen (Wako Pure Chemical, Osaka, Japan) and the first-strand cDNA was synthesized using PrimeScript^®^ 1st strand cDNA Synthesis Kit (Takara Bio Inc., Otsu, Japan), according to the manufacturer’s instructions. Quantitative real-time polymerase chain reaction (PCR) was performed in a volume of 20 μl using gene-specific primers and FastStart Essential DNA Green Master (Roche Applied Science, Branford, CT, USA) in a LightCycler^®^ Nano System (Roche Applied Science). The primer sequences for amplification are shown in Table 1. Reaction efficiency with each primer set was determined using standard amplifications. Target gene expression levels and that of hypoxanthine-guanine phosphoribosyltransferase (HPRT) as a reference gene were calculated for each sample using the reaction efficiency. The results were analyzed using a relative quantification procedure and illustrated as relative expression compared with HPRT expression.

**Table 1:** Primers for real-time PCR.

	Sense primer (5’–3’)	Antisense primer (5’–3’)
IFN-α	TCTGATGCAGCAGGTGGG	AGGGCTCTCCAGACTTCTGCTCTG
IFN-β	GCACTGGGTGGAATGAGACT	AGTGGAGAGCAGTTGAGGACA
iNOS	AGGGAATCTTGGAGCGAGTTGT	GCAGCCTCTTGTCTTTGACCC
MUC2	CAAATCAGGTGGCAGTGTGTTGCT	TGGTAGGAGGAGGGTTGGAAGATG
MUC5AC	ACACCGCTCTGATGTTCCTCACC	ATGTCCTGGGTTGAAGGCTCGT
MUC5B	GGATGGGCAGCAGAAACTGGA	GACAGTGATAGGTGGGATGAAGGTG
Cathelicidin	GACACCAATCTCTACCGTCTCCT	TGCCTTGCCACATACAGTCTCCT
β1-defensing	GAGCATAAAGGACGAGCGA	CATTACTCAGGACCAGGCAGA
S100A8	ACAAGGAAATCACCATGCCCTCTAC	ATGCCACACCCACTTTTATCACCA
S100A9	CAACATCTGTGACTCTTTAGCCTTG	ACTGTGCTTCCACCATTTGTCT
HPRT	GCTTCCTCCTCAGACCGCTT	TCGCTAATCACGACGCTGGG

iNOS: inducible nitric oxide synthase, HPRT: hypoxanthine-guanine phosphoribosyltransferase.

### Preparation of lung leukocytes

Pulmonary intraparenchymal leukocytes were prepared as previously described [31]. Briefly, the chest of the mouse was opened and the lung vascular bed was flushed by injecting 3 ml of chilled physiological saline into the right ventricle. The lungs were then excised and washed in physiological saline. The lungs, teased apart with a 40 μm cell strainer (BD Falcon, Bedford, MA, USA), were incubated in RPMI1640 medium (Nipro, Osaka, Japan) with 5% fetal calf serum (FCS; BioWest, Nuaillé, France), 100 U/ml penicillin G, 100μg/ml streptomycin, 10 mM 4-(2-hydroxyethyl)-1-piperazineethanesulphonic acid (HEPES), 50 μM 2-mercapto ethanol, and 2 mM L-glutamine containing 20 U/ml collagenase and 1 μg/ml DNase I (Sigma-Aldrich, St. Louis, MO, USA). After incubation for 60 min at 37°C with vigorous shaking, the tissue fragments and the majority of dead cells were removed by passing through the 40 μm cell strainer. After centrifugation, the cell pellet was resuspended in 4 ml of 40% (v/v) Percoll (Pharmacia, Uppsala, Sweden), and layered onto 4 ml of 80% (v/v) Percoll. After centrifugation at 600 *g* for 20 min at 15°C, the cells at the interface were collected, washed three times, and counted using a haemocytometer. The obtained cells were centrifuged onto a glass slide at 110 *g* for 3 min using Cytofuge-2 (Statspin Inc., Norwood, MA, USA), stained with Diff-Quick (Sysmex, Kobe, Japan), and observed under a microscope. The number of leukocyte fractions was estimated by multiplying the total leukocyte number by the proportion of each fraction in 200 cells.

### In vitro stimulation of lymph node cells

Paratracheal lymph node (LN) cells were prepared on day 7 after infection with *C*. *neoformans* and cultured at a concentration of 2×10^6^/ml with various doses of viable yeast cells or concanavalin A (Con A; Sigma-Aldrich, St. Louis, MO, USA), as a positive control, in RPMI1640 medium (Nipro, Osaka, Japan) supplemented with 10% FCS (BioWest, Nuaillé, France), 100 U/ml penicillin G, 100 μg/ml streptomycin and 50 μM 2-mercaptoethanol (Sigma-Aldrich) in a 5% CO2 incubator for 48 h. The culture supernatants were collected and stored at -70°C before use.

### Intracellular staining of IL-4

The lung leukocytes and paratracheal LN cells obtained from WT and IFNAR1KO mice on day 7 after infection with *C*. *neoformans* were cultured at 1 × 10^6^/ml with 5 ng/ml phorbol 12-myristate 13-acetate (PMA), 500 ng/ml ionomycin, and 2 nM monensin (Sigma-Aldrich) in RPMI1640 medium supplemented with 10% FCS for 4 h. The cells were washed three times in phosphate-buffered saline (PBS) containing 1% FCS and 0.1% sodium azide and then stained with allophycocyanin (APC)-anti-CD3ε mAb (clone 145-2C11, Biolegend) and phycoerythrin (PE)-anti-CD4 mAb (clone GK1.5, Biolegend). After washing twice, the cells were incubated in the presence of cytofix/cytoperm (BD Bioscience), washed twice in BD perm/wash solution (BD Bioscience), and stained with Alexa Fluor 488-anti-IL-4 (clone 11B11, Biolegend). Isotype-matched IgG was used for control staining. The stained cells were analyzed using a BD FACS Canto^TM^ II flow cytometer (BD Bioscience). Data were collected from 20,000 to 30,000 individual cells using forward-scatter and side-scatter parameters to set a gate on the lymphocyte or myeloid cell population. The number of IL-4-expressing cells was estimated by multiplying the lymphocyte or myeloid cell number, calculated as mentioned above, by the proportion of each subset.

### Cytokine Assay

Mice were sacrificed on days 3, 7, 14, and 28 after infection, and the lungs were excised and then homogenized separately in 5 ml of PBS by teasing through a stainless-steel mesh. After centrifugation, the supernatants were collected and stored at -70°C before use. Concentrations of IFN-γ, IL-12p70, IL-17A, IL-23p19, IL-4, IL-5, and IL-13 in the lung homogenates were measured using each enzyme-linked immunosorbent assay (ELISA) kit (Biolegend for IFN-γ, IL-12p70, IL-17A, IL-4, and IL-5 and eBioscience, San Diego, CA, USA for IL-23p19 and IL-13). IFN-γ, IL-17A, and IL-4 concentrations in the *in vitro* culture supernatants, prepared as mentioned above, were also measured. The detection limit was 4 pg/ml for IFN-γ, 4 pg/ml for IL-12p70, 8 pg/ml for IL-17A, 8 pg/ml for IL-23p19, 1 pg/ml for IL-4, 4 pg/ml for IL-5, and 2.8 pg/ml for IL-13.

### Statistical analysis

Data were analyzed using JMP^®^ Pro 11. 2. 0 software (SAS Institute Japan, Tokyo, Japan). Data are expressed as mean ± standard deviation (SD). Differences between groups were examined for statistical significance using Welch’s *t*-test for two groups and analysis of variance (ANOVA) and the Tukey Kramer test for more than three groups. A *p* value less than 0.05 was considered significant.

## Results

### Effect of IFNAR1 deficiency on the host defense to *C*. *neoformans* infection

**Fig 1:**
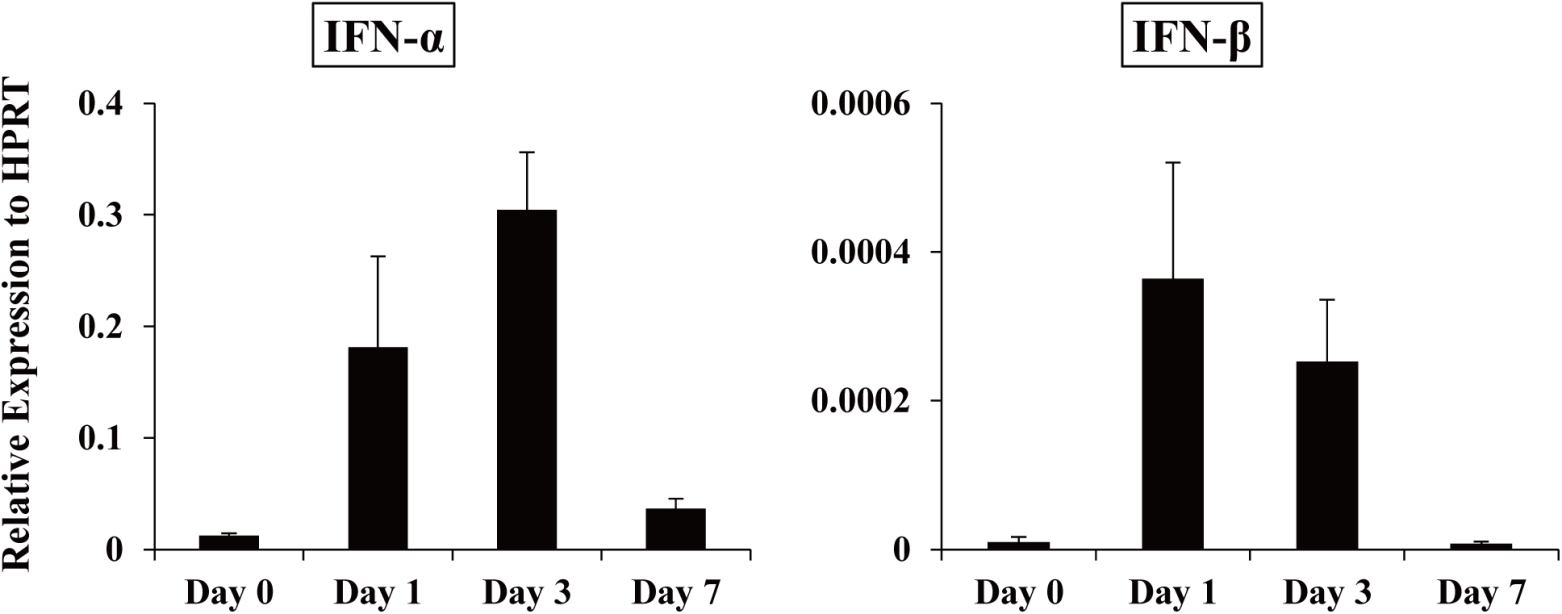
Kinetics in the expression of type I IFNs in the lungs after infection with *C*. *neoformans*. WT mice were infected intratracheally with *C*. *neoformans*. Expression of IFN-α and IFN-β mRNA in the lungs was measured on day 0, 1, 3, and 7 after infection. Each column represents the mean ± SD of three to six mice. Experiments were repeated twice with similar results and the representative data are shown.

**Fig 2:**
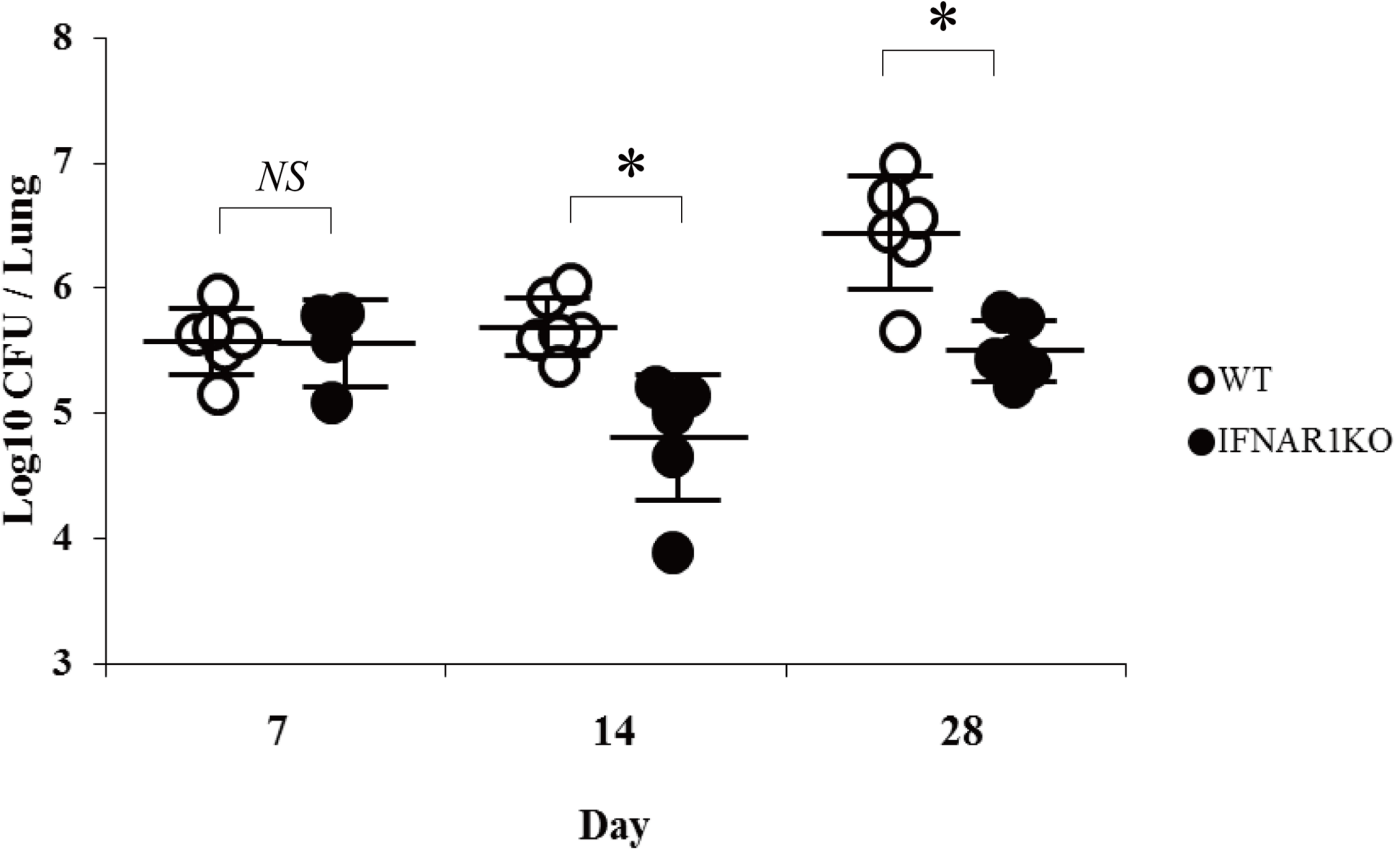
*C*. *neoformans* infection in IFNAR1KO mice. WT and IFNAR1KO mice were infected intratracheally with *C*. *neoformans*. The number of live colonies in the lungs was counted on day 7, 14, and 28 after infection. Each symbol represents each mouse and bars indicate the mean ± SD of five to six mice. Experiments were repeated twice with similar results and the representative data are shown. *NS*, not significant; *, *p < 0*.*05*.

First, to examine whether type I IFN is produced by *C*. *neoformans* infection, we measured the expression of IFN-α and IFN-β at the mRNA level in the lungs at various time intervals after infection with this fungal pathogen. As shown in Fig 1, both IFN-α and IFN-β mRNA expression reached peak levels in an early phase (on day 1 or day 3) and then quickly returned to the basal level on day 7. These results raised a possibility that type I IFN may play some role in the host defense to cryptococcal infection. To address this possibility, IFNAR1KO mice, which have a defect in signaling caused by type I IFNs, were infected with *C*. *neoformans*, and the growth of this fungal pathogen in the lungs was examined. As shown in Fig 2, the number of live colonies was equivalent on day 7 between WT and IFNAR1KO mice and significantly lower in IFNAR1KO mice than in WT mice on day 14 and 28 post-infection. The live colonies were not found in the brains of WT and IFNAR1KO mice at these time points, and all the mice did not die during the observation periods.

### Increased Th1 response in IFNAR1KO mice

**Fig 3:**
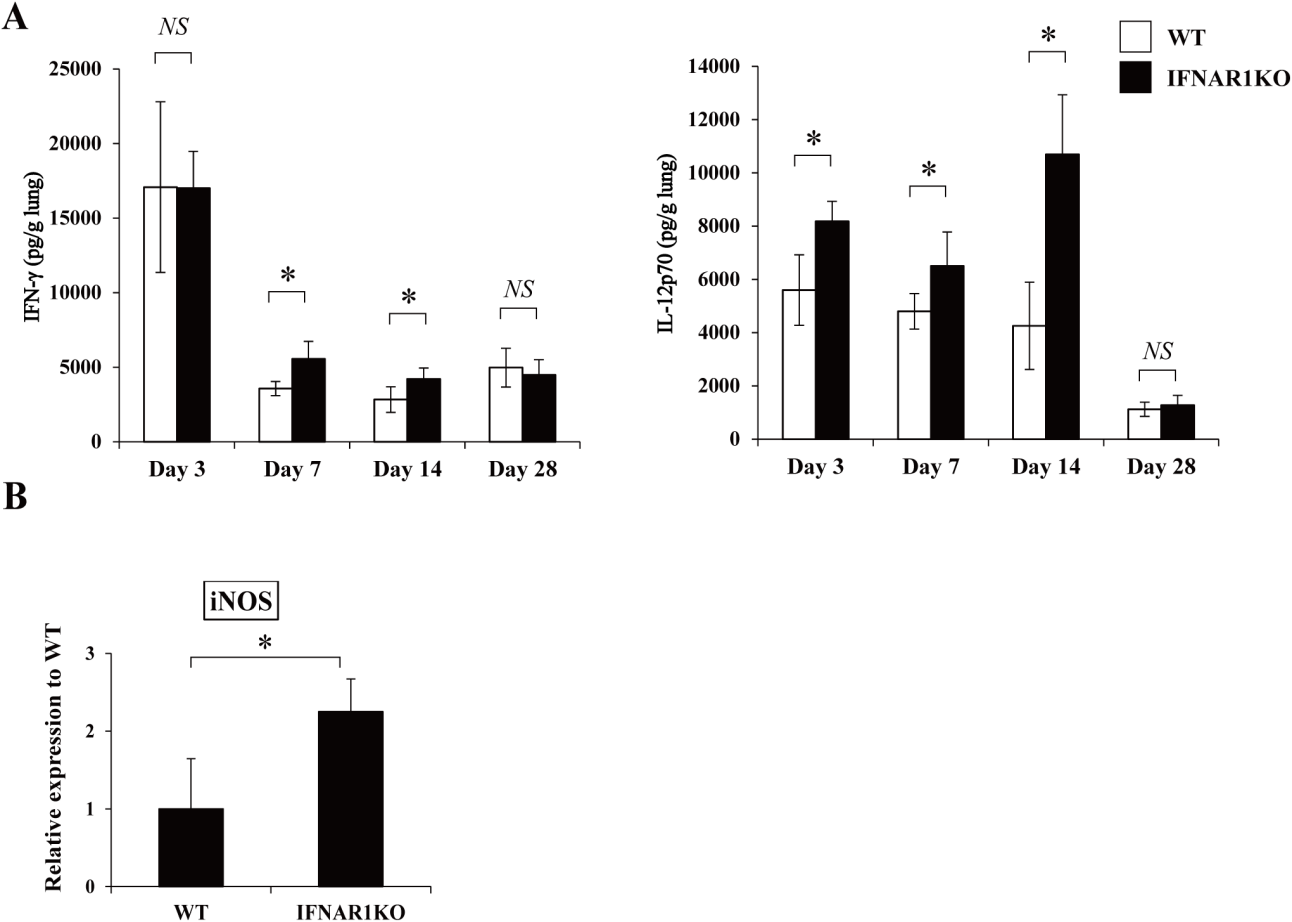
Effect of IFNAR1 deficiency on the Th1-related response. WT and IFNAR1KO mice were infected intratracheally with *C*. *neoformans*. (***A***) IFN-γ and IL-12p70 production in the lung homogenates was measured on day 3, 7, 14, and 28. Each column represents the mean ± SD of five to six mice. (***B***) Expression of iNOS mRNA in the lungs was measured on day 7 after infection. Each column represents the mean ± SD of five mice. Experiments were repeated twice with similar results and the representative data are shown. *NS*, not significant; *, *p < 0*.*05*.

It has been well documented that the Th1 response is essential for the host defense to *C*. *neoformans* infection [2, 12]. To clarify how a defect in type I IFN signaling affects the Th1 response, production of IFN-γ and IL-12p70, a critical cytokine for IFN-γ production, in the lungs was compared between WT and IFNAR1KO mice. As shown in Fig 3A, IFN-γ and IL-12p70 were produced at a significantly higher level in IFNAR1KO mice than in WT mice on day 7 and 14 and on day 3, 7 and 14 post-infection, respectively.

Promotion of the Th1 response leads to acceleration in clearing *C*. *neoformans* via increased NO synthesis by macrophages that are triggered by IFN-γ stimulation [10, 18, 19, 32]. The NO-dependent macrophage killing is essential for the host defense to this fungal pathogen [10, 18, 19, 32]. Therefore, we measured the expression of iNOS at a mRNA level in the lungs infected with *C*. *neoformans*. As shown in Fig 3B, IFNAR1KO mice expressed a significantly higher level of iNOS mRNA than WT mice.

### Effect of IFNAR1 deficiency on Th17 response

**Fig 4:**
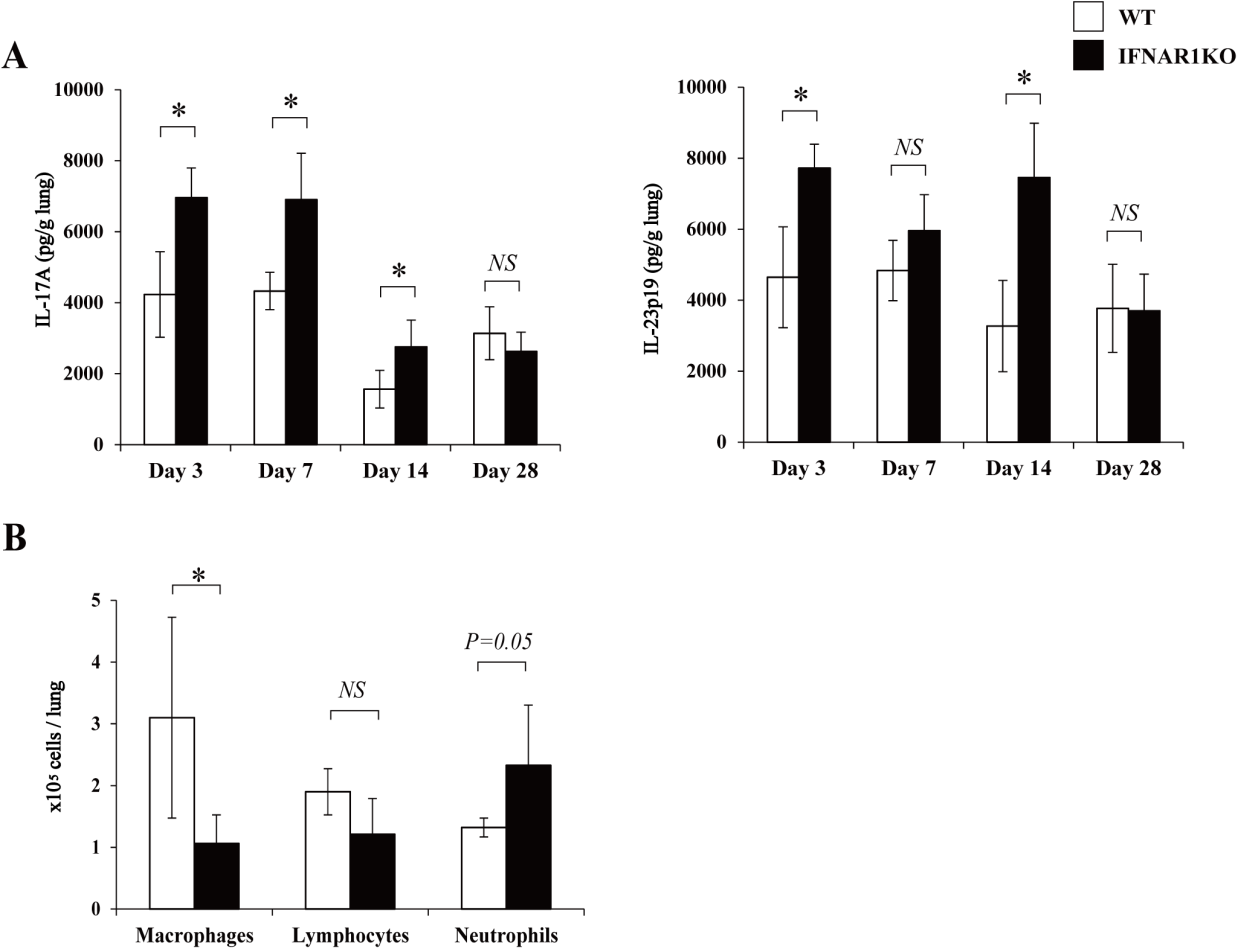
Effect of IFNAR1 deficiency on the Th17-related response. WT and IFNAR1KO mice were infected intratracheally with *C*. *neoformans*. (***A***) IL-17A and IL-23p19 production in the lung homogenates was measured on day 3, 7, 14, and 28. Each column represents the mean ± SD of five to six mice. (***B***) The lung leukocytes prepared on day 7 post-infection were stained with Diff-Quick and observed under a light microscope. The amount of cells in each leukocyte fraction was counted. Each column represents the mean ± SD of five mice. Experiments were repeated twice with similar results and the representative data are shown. *NS*, not significant; *, *p < 0*.*05*.

In the next experiment, we examined how the deficiency of type I-IFN signaling affected the Th17 response to cryptococcal infection by measuring the production of IL-17A and IL-23p19 in the lungs. As shown in Fig 4A, production of IL-17A and IL-23p19 was significantly increased on day 3, 7 and 14 and on day 3 and 14, respectively, in IFNAR1KO mice compared to WT mice.

Because IL-17A is known to play an important role in the neutrophilic inflammatory response [33], we counted the number of neutrophils accumulated in the lungs infected with *C*. *neoformans*. As shown in Fig 4B, the number of neutrophils tended to be increased in IFNAR1KO mice compared to WT mice, although the difference was not statistically significant. In contrast, macrophages were significantly reduced in IFNAR1KO mice compared to WT mice.

### Effect of IFNAR1 deficiency on Th2 response

**Fig 5:**
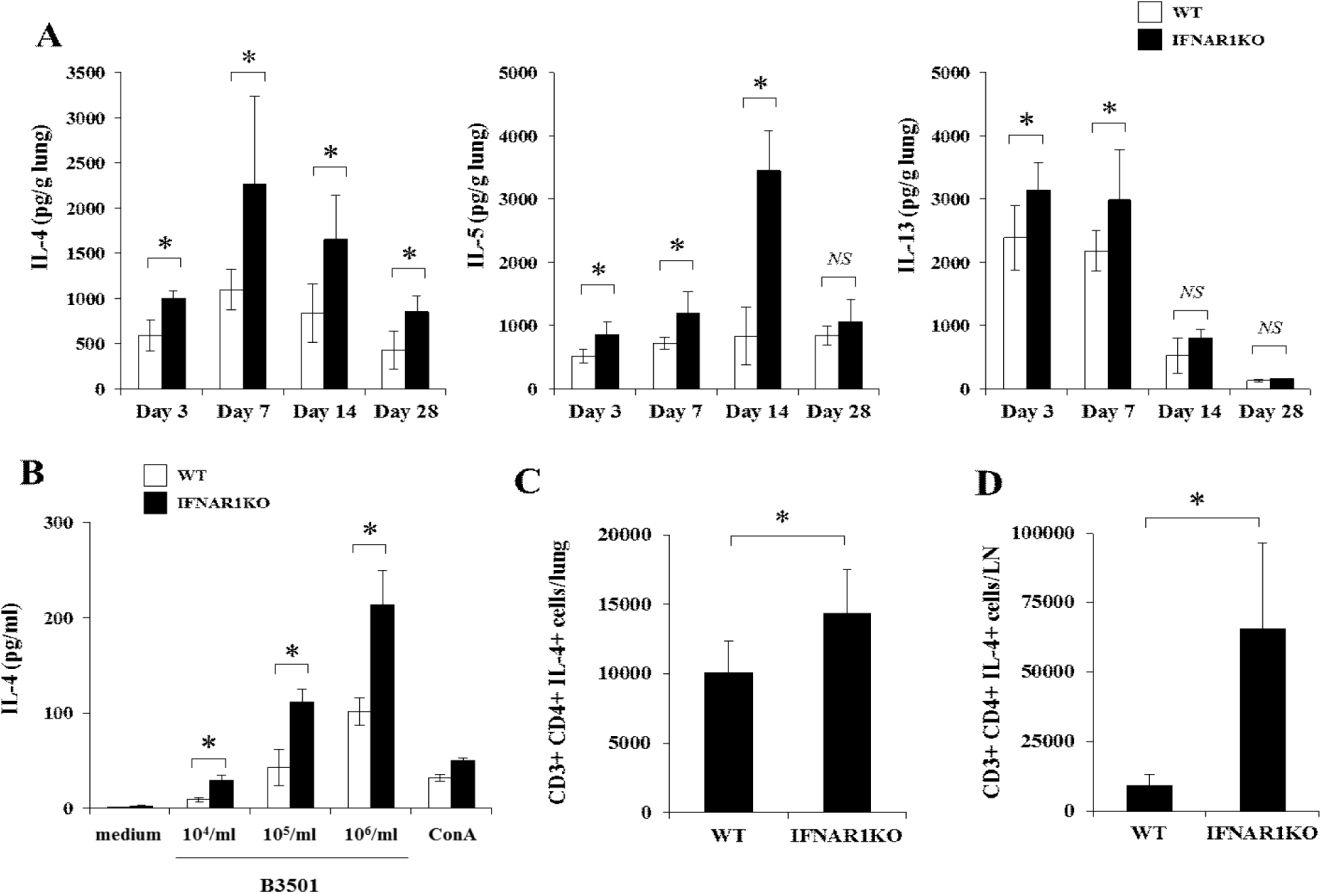
Effect of IFNAR1 deficiency on the Th2-related response. WT and IFNAR1KO mice were infected intratracheally with *C*. *neoformans*. (***A***) IL-4, IL-5, and IL-13 production in the lung homogenates was measured on day 3, 7, 14, and 28 after infection. Each column represents the mean ± SD of five to six mice. (***B***) LN cells obtained on day 7 post-infection were stimulated with indicated doses of *C*. *neoformans* or ConA for 48 h, and production of IL-4 was measured. Each column represents the mean ± SD of triplicate cultures. The lung leukocytes (***C***) and LN cells (***D*)** were prepared on day 7 post-infection. Expression of IL-4 in CD3^+^ CD4^+^ T cells was analyzed using flow cytometry and the number of IL-4^+^ CD3^+^ CD4^+^ T cells was calculated. Each column represents the mean ± SD of five mice. Experiments were repeated twice with similar results and the representative data are shown. *NS*, not significant; *, *p < 0*.*05*.

We further examined the effect of IFNAR1 deficiency on the Th2 response by measuring the production of IL-4, IL-5, and IL-13 in the lungs on day 3, 7, 14, and 28 after cryptococcal infection. As shown in Fig 5A, levels of these cytokines in the lung homogenates were significantly higher in IFNAR1KO mice than in WT mice at these time intervals, except for IL-5 on day 28 and IL-13 on day 14 and 28. These results suggested that differentiation of Th2 cells may be promoted under a condition lacking type I IFN signaling during infection with this fungal pathogen. To address this possibility, IL-4 production by paratracheal LN cells was measured upon re-stimulation with this fungal pathogen. As shown in Fig 5B, LN cells from IFNAR1KO mice produced a significantly higher amount of IL-4 than those from WT mice. In addition, in a flow cytometric analysis, we examined the intracellular expression of IL-4 by CD4^+^T cells in the lungs and paratracheal LN infected with *C*. *neoformans*. The number of IL-4-expressing cells was significantly increased in IFNAR1KO mice compared to WT mice (Fig 5C and 5D).

### Increased production of mucin in IFNAR1KO mice

**Fig 6:**
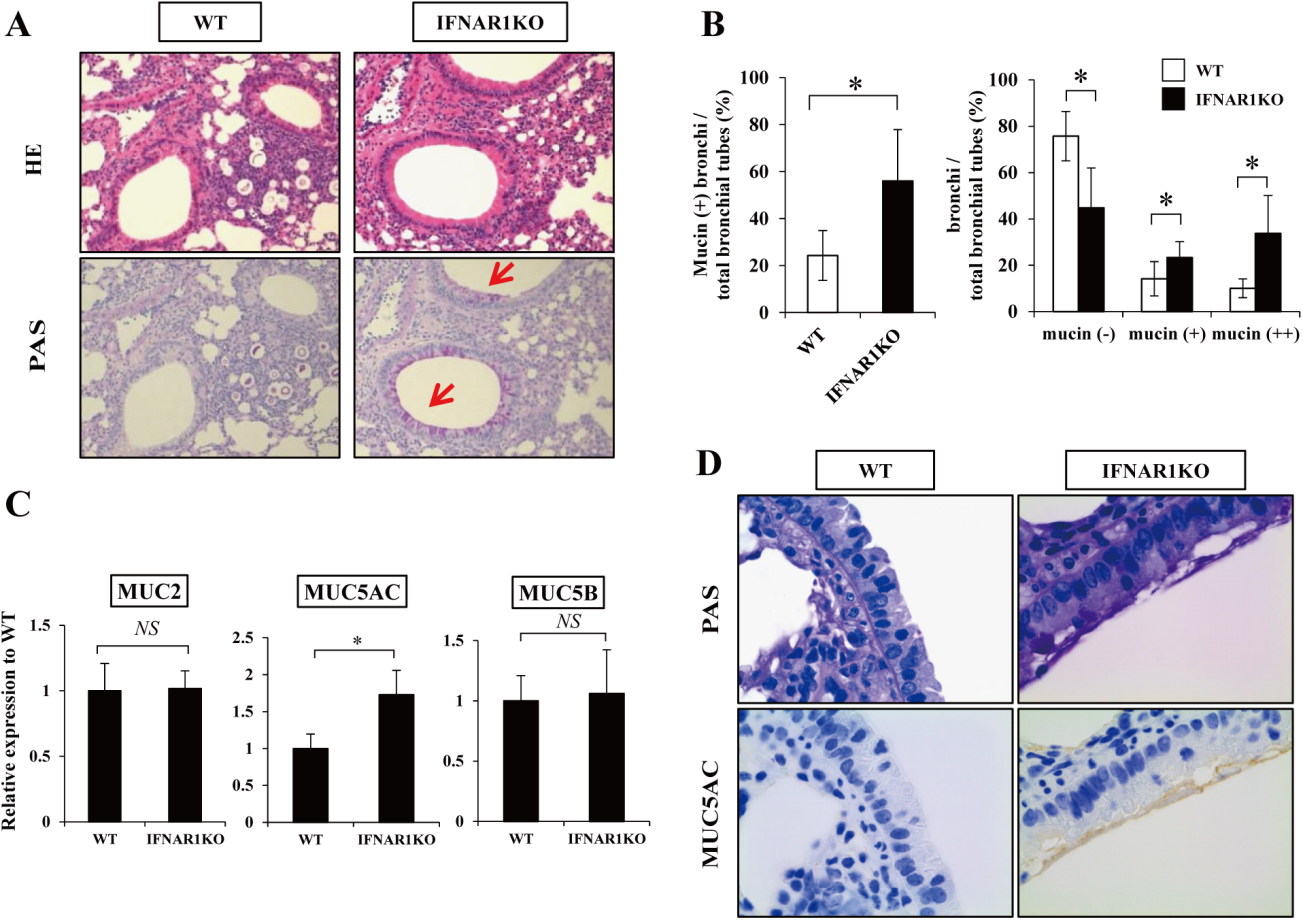
Mucin production after infection with *C*. *neoformans*. WT and IFNAR1KO mice were infected intratracheally with *C*. *neoformans*. (***A***) Sections of the lungs on day 14 post-infection were stained with H-E or PAS and observed under a light microscope at ×200. Representative pictures of six mice are shown. Red arrows show the mucin production. (***B***) The proportion of mucin-producing bronchi (left panel) and the classification of mucin-producing bronchi (right panel) was calculated. Each column represents the mean ± SD of six mice. Experiments were repeated twice with similar results and the representative data are shown. (***C***) Expression of MUC2, MUC5AC, and MUC5B mRNA in the lungs was measured on day 7 after infection. Each column represents the mean ± SD of five mice. Experiments were repeated twice with similar results and the representative data are shown. (***D***) Sections of the lungs on day 14 post-infection were immunohistochemical stained with MUC5AC and observed under a light microscope at ×1000. MUC5AC expression is shown in brown. Representative pictures of six mice are shown. *NS*, not significant; *, *p < 0*.*05*.

Previously, Th2 cytokines, such as IL-4 and IL-13, were found to promote the production of mucin in the lungs infected with *Pseudomonas aeruginosa* and *C*. *neoformans* and lead to improved clearance [34–37]. In the next series of experiments, therefore, we focused on the effect of IFNAR1 deficiency on mucin production after infection with *C*. *neoformans*. First, we evaluated the bronchoepithelial cells expressing mucin in the PAS-stained lung specimens. As shown in Fig 6A, the proportion of mucin-producing bronchi was significantly increased in IFNAR1KO mice compared to WT mice on day 14. In addition, the proportion of mucin-negative bronchi was significantly reduced and the proportion of mucin + and ++ bronchi was significantly increased in IFNAR1KO mice compared to WT mice (Fig 6B), indicating the increase in mucin-producing bronchoepithelial cells in IFNAR1KO mice.

There is a variety of mucin consisting of different core proteins, such as MUC1, MUC2, MUC3A, MUC3B, MUC4, MUC5AC, MUC5B, MUC6, and so on [38]. Among them, MUC2, MUC5AC, and MUC5B are major core proteins forming the mucin secreted in the lungs [38, 39]. Thus, next, we examined the expression of MUC2, MUC5AC, and MUC5B mRNA in the lungs infected with *C*. *neoformans*. As shown in Fig 6C, MUC5AC expression was significantly increased in IFNAR1KO mice compared to that in WT mice, whereas expression of MUC2 and MUC5B was comparable between these mouse strains. In addition, in an immunohistochemical analysis, bronchoepithelial cells expressing MUC5AC were increased in IFNAR1KO mice compared to WT mice (Fig 6D).

Type I IFNs were reported to suppress the production of antimicrobial peptides during infection with *Mycobacterium leprae* [40]. Thus, we examined the lung expression of antimicrobial peptides, such as cathelicidin, β1-defensin, and S100A8/9, at the mRNA level after cryptococcal infection. In the current study, these molecules were expressed at an equivalent level between WT and IFNAR1KO mice (S1 Fig).

### IL-4-dependent increase of mucin production in IFNAR1KO mice

**Fig 7:**
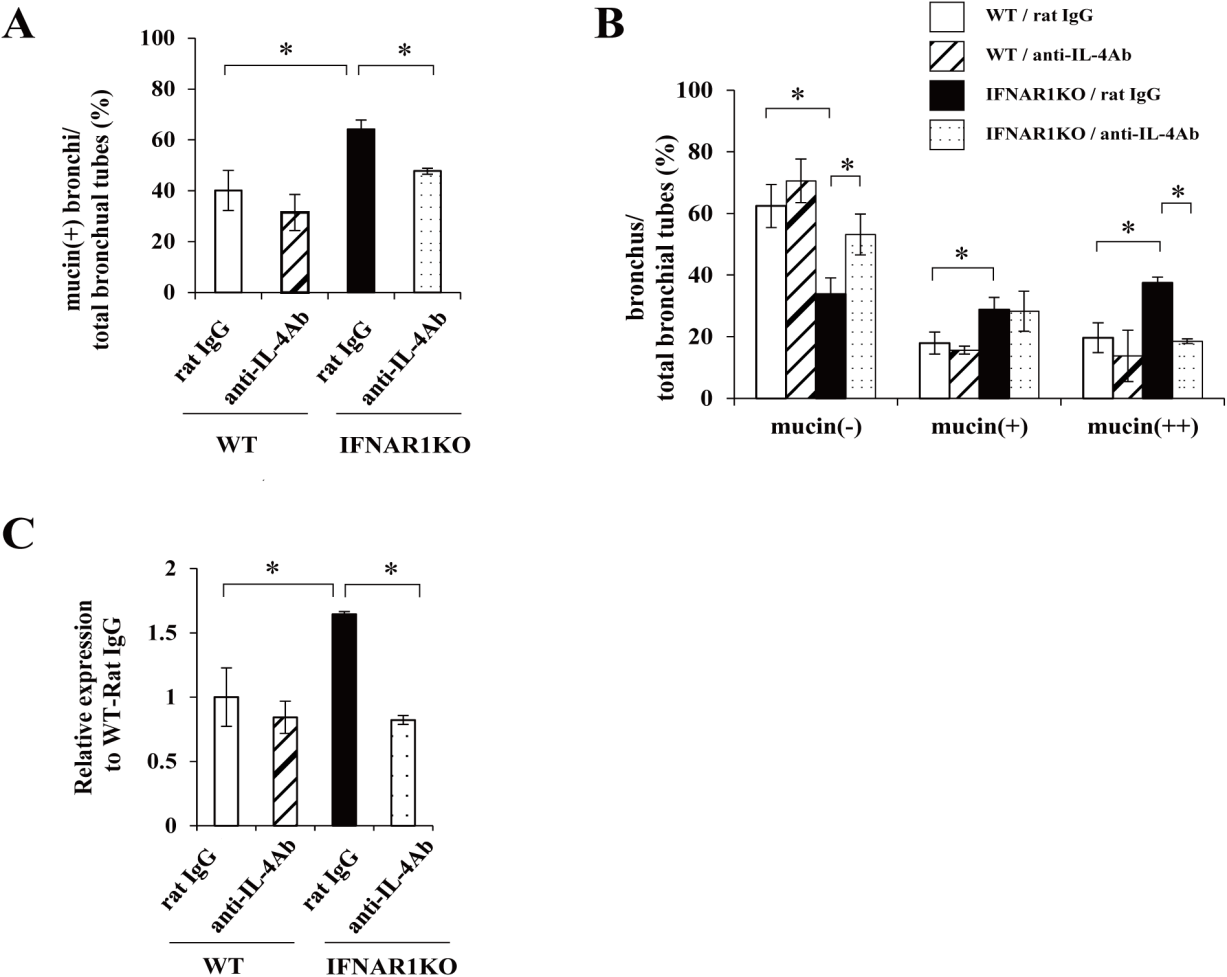
Effect of anti-IL-4 mAb on mucin production. WT and IFNAR1KO mice were infected intratracheally with *C*. *neoformans*. Mice were injected intraperitoneally with anti-IL-4 mAb and control rat IgG. Sections of the lungs on day 14 post-infection were stained with H-E or PAS and observed under a light microscope. The proportion of mucin-producing bronchi (***A***) and the classification of mucin-producing bronchi (***B***) was calculated. Each column represents the mean ± SD of three mice. (***C***) Expression of MUC5AC mRNA in lungs was measured on day 7 after infection. Each column represents the mean ± SD of three mice. Experiments were repeated twice with similar results and the representative data are shown. *, *p < 0*.*05*.

To clarify the relationship between mucin and IL-4, the production of which was increased in IFNAR1KO mice, we examined the effect of neutralizing anti-IL-4 mAb on the mucin production and the expression of MUC5AC mRNA in lungs infected with *C*. *neoformans*. As shown in Fig 7A, the mucin-producing bronchi was significantly higher in IFNAR1KO mice treated with rat IgG than in WT mice treated with rat IgG, and treatment with anti-IL-4 mAb led to a significant reduction in mucin production in IFNAR1KO mice, but not in WT mice. Similar results were obtained in the mucin-producing bronchoepithelial cells and in the expression of MUC5AC mRNA (Fig 7B and 7C).

### Suppression of IL-4 synthesis by rIFN-αA/D

**Fig 8:**
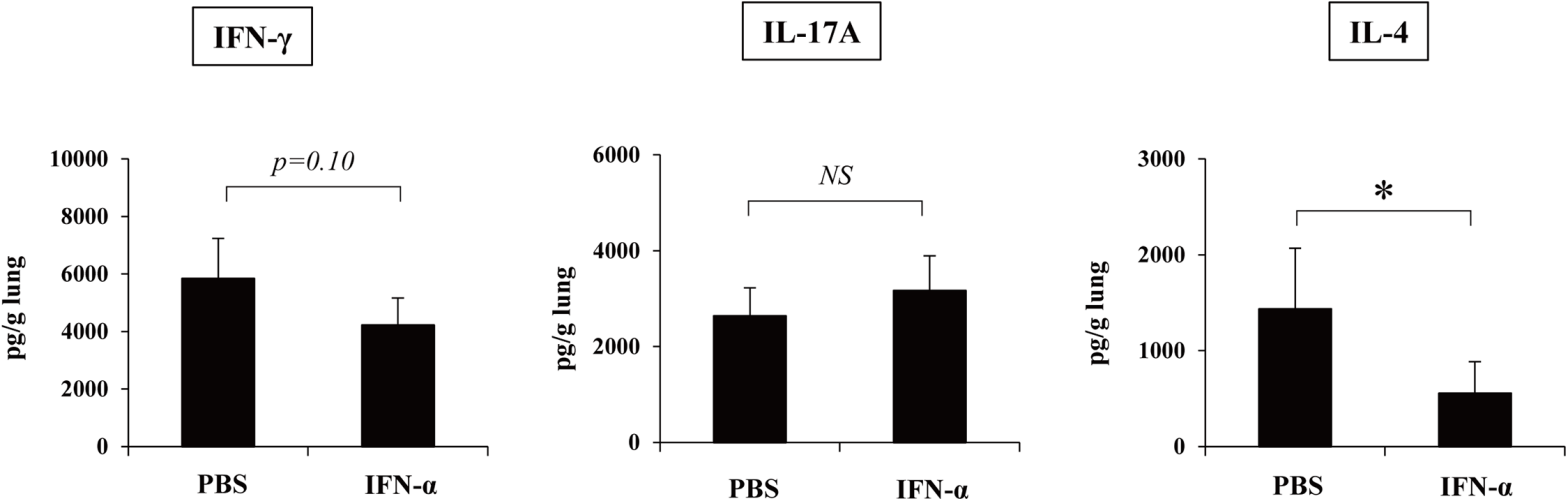
Effect of rIFN-αA/D on IFN-γ and IL-4 synthesis. C57BL/6 mice were infected intratracheally with *C*. *neoformans*. Mice received daily administration of 1,000 IU/mouse rIFN-αA/D after infection. IFN-γ, IL-17A, and IL-4 production in the lungs was measured on day 14 after infection. Each column represents the mean ± SD of six mice. *NS*, not significant; *, *p* < 0.05.

In the current study, to address the role of type I IFN in the host defense to cryptococcal infection, we used IFNAR1KO mice, which showed an increased Th1, Th2, and Th17 response in the infected lungs. In the subsequent experiments, we examined how this infection was affected by the administration of type I IFN. As shown in Fig 8, the administration of rIFN-αA/D significantly reduced the production of IL-4, but not of IFN-γ and IL-17A, in the lungs on day 14.

## Discussion

In the current study, we demonstrated that type I IFNs were quickly expressed in an innate immune phase after infection with *C*. *neoformans*. Type I IFNs are produced by almost all cells via various pattern recognition receptors, including the retinoic acid–inducible gene I (RIG-I), melanoma differentiation-associated gene 5 (MDA5), and toll-like receptors (TLRs) 3, 4, 7, and 9 [41]. Plasmacytoid dendritic cells (pDCs) are known to produce a large amount of type I IFNs upon viral infection through the TLR7 and TLR9 system [42]. Recently, Ramirez-Ortiz and co-workers reported that pDCs released IFN-α via a TLR9-independent mechanism after stimulation with *Aspergillus fumigatus* [43]. In addition, del Fresno and co-workers reported that type I IFNs were produced by BM-DCs upon stimulation with *C*. *albicans* via Dectin-1 and Dectin-2 [44]. Thus, type I IFNs may be produced by a variety of cells, including pDCs and myeloid DCs, after infection with *C*. *neoformans*, although the details remain to be elucidated.

The number of live colonies in the lungs was significantly reduced in the early phase of infection with *C*. *neoformans* in IFNAR1KO mice compared to WT mice. These data suggest that type I IFNs may have detrimental effects on the early host defense response to cryptococcal infection. Supporting this possibility, the production of Th1-related cytokines such as IFN-γ and IL-12p70 and the expression of iNOS, which is critical for eradication of this infection [12], were enhanced in IFNAR1KO mice. Here, we examined the expression of iNOS mRNA in the lung homogenates, which does not necessarily mean that this expression was exclusively derived from macrophages. Previous investigations also demonstrated the type I IFN-induced suppression of IFN-γ in viral and bacterial infections [45, 46].

Type I IFNs improve experimental autoimmune encephalomyelitis by suppressing the Th17 response and are used for the treatment of multiple sclerosis in clinical settings [47, 48]. In the current study, the production of IL-17A and IL-23p19 in the lungs was higher in IFNAR1KO mice than in WT mice after cryptococcal infection, suggesting that the promoted Th17 and neutrophilic response may account for the increased host resistance to cryptococcal infection in IFNAR1KO mice. Consistent with this, neutrophils were shown to kill *C*. *neoformans* and to promote host defense [49, 50], although other studies revealed that their presence did not correlate with the protective response to this infection [51–53]. In our recent study, the lung clearance of *C*. *neoformans* was not hampered in IL-17AKO mice compared to WT mice [54], suggesting that the neutrophilic response may not be involved in the protection against this fungal pathogen. In contrast, other studies reported the important role of IL-17A in the host defense to *C*. *neoformans* [55, 56]. Further investigations are necessary to determine the precise role of Th17 and the neutrophilic response.

Host defense to cryptococcal infection is thought to be critically regulated by a balance between the Th1 and Th2 response [2, 3, 12]. A shift of this balance toward the Th2-dominated response results in worsening of this infection [12, 57]. However, in the current study, both Th1 and Th2 responses were elevated in the infected lungs of IFNAR1KO mice, suggesting the involvement of a more complex mechanism. Interestingly, Leibundgut-Landmann and co-workers demonstrated that Th2 cytokines contribute to the elimination of *P*. *aeruginosa* by bronchoepithelial cells through promoting the production of mucin and anti-microbial peptides [34]. Furthermore, Grahnert et al. recently reported that IL-4RαKO mice showed attenuated control of *C*. *neoformans* infection with decreased cellular inflammatory response and reduced mucin production in the lungs compared to WT mice [35]. In line with these earlier studies, in our study, mucin production and expression of MUC5AC by bronchoepithelial cells were promoted in the lungs of IFNAR1KO mice after infection with *C*. *neoformans*, although the mucin-producing bronchi was not detected in naïve IFAR1KO mice or WT mice (data not shown). In addition, this increased mucin production was totally dependent on IL-4. Mucin makes an important contribution to the mechanical clearance of microorganisms via a mucociliary escalator system [58], suggesting that mucin production could be one of the mechanisms for the decreased number of live *C*. *neoformans* in IFNAR1KO mice. In agreement with this possibility, Hasnain and co-workers recently demonstrated that mice lacking MUC5AC are susceptible to enteric infection with *Trichuris muris* [59]. In another study, type I IFNs were reported to suppress the production of antimicrobial peptides during infection with *Mycobacterium leprae* [40]. However, in our study, antimicrobial peptides such as cathelicidin, β1-defensin, and S100A8/9 were not increased in the lungs of IFNAR1KO mice after cryptococcal infection (S1 Fig). At the moment, it remains unclear whether the clearance of *C*. *neoformans* can be accounted for by mucin production without an increase in antimicrobial peptides.

Treatment with rIFN-αA/D resulted in significantly reduced production of IL-4, but not of IFN-γ and IL-17A, on day 14 after infection with *C*. *neoformans* (Fig 8), which was consistent with our hypothesis raised by the current data with IFNAR1KO mice suggesting that type I IFNs may have detrimental effects on the early host defense to this infection by suppressing the Th1 and Th2 immune response. However, administration of rIFN-αA/D did not affect the clearance of *C*. *neoformans* and mucin production in the lungs (data not shown). Some reports showed that rIFN-α was degraded rapidly and became undetectable within 24 h after administration [60–62]. However, 2'-5' oligoadenylate synthetase, which is induced by type I IFN signaling, peaked at 12 h and was detected 48 h after rIFN-α administration [60, 61]. Therefore, the biological effects of type I IFN signaling would continue for a considerable time even after rIFN-α was undetectable. On the basis of these findings, in the current study, rIFN-αA/D was administered once a day in mice. However, the possibility cannot be excluded that rIFN-αA/D might produce distinct effects by shortening the administration interval.

Although the effects of type I IFNs administration on bacterial and fungal infection has not been clarified, treatment with its agonists such as poly(I:C) was reported to show detrimental effects during bacterial infection [63, 64]. Administration of poly(I:C) impaired the clearance of *M*. *tuberculosis* and *Streptococcus pneumonia* in the lungs, which was dependent on IFNAR signaling. It remains to be addressed in the current study why the number of live *C*. *neoformans* and the synthesis of cytokines in the lungs were differentially affected by the deletion of IFNAR signaling and treatment with rIFN-αA/D. In earlier studies, different signaling and distinct biological effects mediated by IFN-α and IFN-β were reported [65–67], which might be related to the possible reason for the inconsistent results described above: deletion of IFNAR signaling leads to shutoff in triggering not only by IFN-α but also by IFN-β, unlike administration of rIFN-αA/D alone. Alternatively, IFNAR2, another receptor for type I IFNs [22], might be involved in the response to exogenously administered rIFN-αA/D. Further investigation will be necessary to resolve this issue.

Several investigations addressing the significance of type I IFNs in microbial infection [27, 28, 68, 69] are consistent with the current hypothesis that type I IFNs negatively regulate the host defense to infection with *C*. *neoformans*. IFNAR1KO mice were less susceptible to infection with *L*. *monocytogenes*, *Fancisella novicida*, *Mycobacterium tuberculosis*, *Salmonella enterica*, and *Yersinia pestis*, as shown by prolonged survival [28]. The clearance of *L*. *monocytogenes*, *F*. *novicida*, *M*. *tuberculosis*, *S*. *enterica*, *Y*. *pestis*, *Chlamydia muridarum*, *Histoplasma capsulatum*, and *Candida glabrata* was also accelerated in these mice [28, 68, 69]. A highly virulent strain of *Mycobacterium tuberculosis* failed to stimulate a Th1-type response, which was associated with increased induction of type I IFNs synthesis [27]. However, these studies did not focus on how type I IFNs act on the Th1-Th2 balance and mucin production during the infection.

In conclusion, the current study demonstrated that the clearance of *C*. *neoformans* in the lungs was accelerated at the early phase of infection, with the increase of not only the Th1 but also the Th2-type response and IL-4-dependent mucin production, under a condition lacking type I IFNs-mediated signaling. These findings suggested that type I IFNs may be involved in negative regulation of the early host defense to this infection by suppressing the Th1-mediated immune response and by mechanical clearance at the mucosal surfaces. Thus, our findings have important implications to our understanding of the pathogenic mechanism of infection with *C*. *neoformans*.

## Supporting Information

S1 Fig Effect of IFNAR1 deficiency on anti-microbial peptides. Expression of cathelicidin, β1-defensing and S100A8/9 mRNA in the lungs was measured on day 7 after infection. Each column represents the mean ± SD of five mice. Experiments were repeated twice with similar results. *NS*, not significant.
(TIF)
