# Supplementary figures and images for: Cryptococcus neoformans Infection in Mice Lacking Type I Interferon Signaling Leads to Increased Fungal Clearance and IL-4-Dependent Mucin Production in the Lungs
Source: PLoS One. 2015 Sep 18;10(9):e0138291. doi: 10.1371/journal.pone.0138291 (PMC4575107; doi:10.1371/journal.pone.0138291)

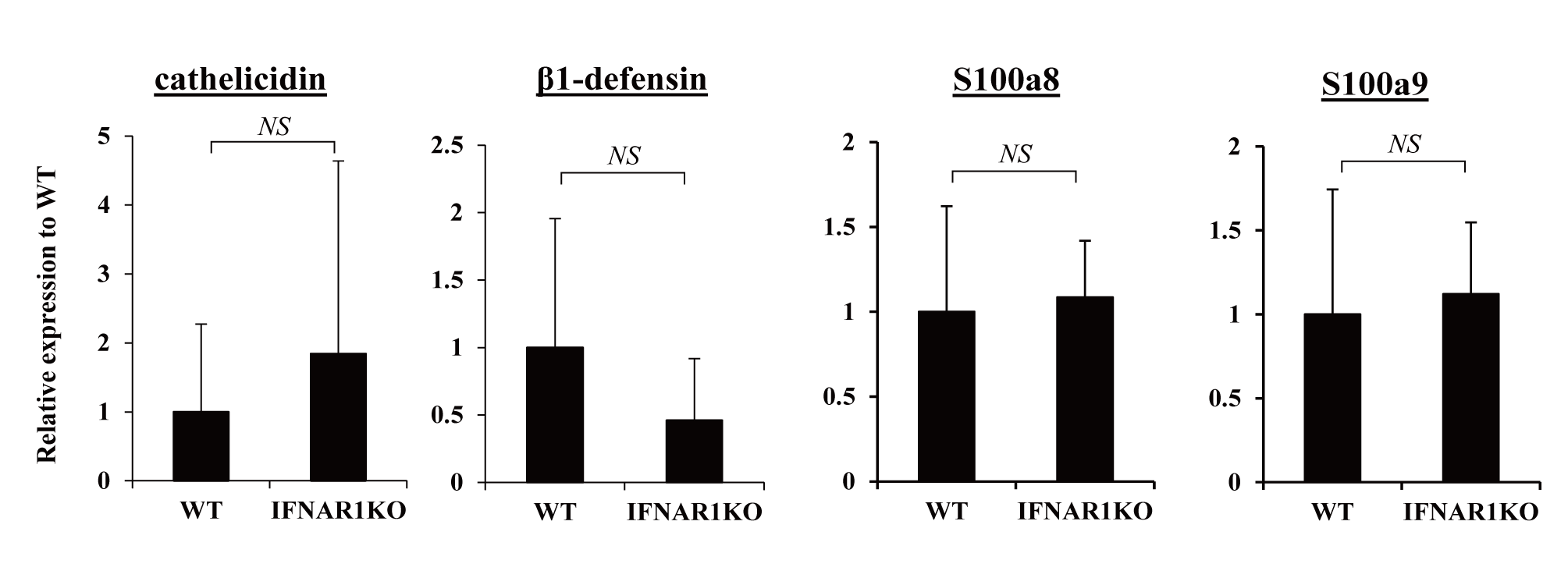

Supplement: S1 Fig — Expression of cathelicidin, β1-defensing and S100A8/9 mRNA in the lungs was measured on day 7 after infection. Each column represents the mean ± SD of five mice. Experiments were repeated twice with similar results. NS, not significant. (TIF) [file pone.0138291.s001.tif]
